# The Influence of Perceived Crowdedness on Aggressive Behavior: A Moderated Mediation Model

**DOI:** 10.3390/bs15030259

**Published:** 2025-02-24

**Authors:** Yue Xie, Wenwu Dai, Zhihui Yang

**Affiliations:** School of Humanities and Social Sciences, Beijing Forestry University, Beijing 100083, China

**Keywords:** aggressive behavior, perceived crowdedness, relative deprivation, upward social comparison, connectedness to nature

## Abstract

Aggressive behavior not only negatively affects an individual’s psycho-social adaptation but also undermines social harmony and stability. This study investigated the relationship between perceived crowdedness and aggression, examining the mediating role of relative deprivation and the moderating effects of upward social comparison and connectedness to nature based on the general strain theory and the I^3^ model. A cross-sectional design was employed, involving 848 participants (524 females, 61.8%) aged 20–75 (*M* = 33.15, *SD* = 6.83) in China. Structural equation modeling and bootstrapping procedures were used to test the hypothesized moderated mediation model. The results indicated that perceived crowdedness and upward social comparison positively predicted aggressive behavior, whereas connectedness to nature negatively predicted it. Relative deprivation mediated the relationship between perceived crowdedness and aggressive behavior. Only upward social comparison moderated the relationship between perceived crowdedness and relative deprivation, and connectedness to nature showed a marginally significant moderating effect on the relationship between perceived crowdedness and aggressive behavior. Unexpected trends are discussed, along with directions for future research.

## 1. Introduction

Aggressive behavior refers to any intentional act aimed at harming another individual, resulting in a negative impact either physically or psychologically (Anderson & Bushman, 2001), which includes forms such as physical aggression, verbal aggression, anger, and hostility (Buss & Perry, 1992). It has negative effects not only on the victims but also on the aggressors, particularly in terms of psycho-social adaptation.

For victims, individuals who experience aggressive behavior are at risk of depression, anxiety, and even suicidal ideation (Gros et al., 2010; Hart & Ostrov, 2020; Loudin et al., 2003). For aggressors, such individuals may develop problematic personality traits, substance abuse, high unemployment rates, and criminal behavior (Dahlen et al., 2013; Piquero et al., 2007). In addition to the negative consequences of aggressive behavior at the individual level, aggressive behavior also undermines social harmony and stability (Bird et al., 2001). Given the huge negative effects of aggressive behavior, understanding the influencing factors and mechanisms behind individual expressions of aggressive behavior holds significant implications for both personal development and societal construction.

Individuals’ behavior is influenced not only by the psychological environment but also by the physical environment. Previous research has primarily focused on exploring the impact of the psychological environment on individual aggressive behavior and its underlying mechanisms while overlooking the role of the perceived physical environment in shaping aggression. According to previous studies (J. Liu et al., 2023; Regoeczi, 2003; Weber et al., 2024), crowdedness, as an example of an individual’s perception of the physical environment, may predict social behavior. However, the relationship between crowdedness and aggressive behavior, along with its underlying mechanisms, has yet to be explored. Therefore, this study will examine the influencing factors and mechanisms of aggressive behavior by considering both individual and environmental factors simultaneously. The general strain theory (Agnew, 2001) and the I^3^ model provide a better understanding of the mechanisms of individual aggressive behaviors (Finkel & Hall, 2018). The general strain theory posits that negative stimuli evoke negative emotions in individuals, which subsequently lead to the development of aggression (Agnew, 2001). The I^3^ model underscores that the likelihood or intensity of individuals’ behavior is primarily influenced by three factors: instigation, impellance, and inhibition. Instigation refers to environmental stimuli that provoke or trigger individuals to engage in certain behaviors; impellance indicates the individual’s inclination to escalate the tendency to execute a behavior; inhibition involves situational factors that strengthen the suppressive effects on instigation and impellance, thereby diminishing the likelihood or intensity of behavior occurrence (Finkel & Hall, 2018).

Consequently, our study investigated the relationship between environmental factors that directly promote behavior (i.e., crowdedness), negative emotions (i.e., relative deprivation), individual factors that reinforce behavioral tendencies (i.e., upward social comparison), and personality factors that inhibit behavioral tendencies (i.e., connectedness to nature) and aggressive behaviors. Specifically, this study constructed a moderated mediation model to examine the relationships among the aforementioned variables.

## 2. Literature Review and Research Hypothesis

### 2.1. Perceived Crowdedness and Aggressive Behavior

According to the general strain theory and the I^3^ model, crowdedness, as an instigation factor, significantly predicts aggressive behavior. Crowdedness can be categorized into objective crowdedness (i.e., density) and subjective crowdedness (i.e., perceived crowdedness). Objective crowdedness is an attribute of the physical environment, whereas subjective crowdedness is a psychological experience (Stokols, 1972). The former is primarily measured by the quantity and size of physical space, whereas the latter is assessed mainly through self-reported measures. Previous researchers have conducted extensive studies on the relationship between objective crowdedness and aggression-related behaviors, such as violent incidents, conflicts, and antisocial behavior. For example, in a prison environment, Megargee (1977) found that the total amount of personal space and the index of population density were significantly correlated with both the number and rate of violations. In a hospital ward setting, Palmstierna et al. (1991) observed that an increased number of patients in a psychiatric acute care unit significantly heightened the likelihood of aggressive behavior. Similarly, Ng et al. (2001) found that the occupancy level was positively associated with the occurrence of two types of violent incidents, including physical and verbal aggression.

In addition to exploring the relationship between objective crowdedness and aggression in specific environments, researchers have also investigated this relationship within housing settings. Welch and Booth (1975) found that residential density had little correlation with three types of aggression (i.e., family aggression, physical punishment, and aggression beyond the household). However, they discovered that subjective crowdedness was significantly correlated with all three indicators of aggression. A more recent study further confirmed that, compared to the relationship between residential density and aggression, subjective crowdedness exhibited a stronger correlation with aggression (Weber et al., 2024). Both of these studies suggest that within housing research, perceived crowdedness appears to be a stronger predictor of aggressive behavior. Weber et al. (2024) highlighted that, given the greater impact of subjective crowdedness on psychological outcomes compared to residential density and the extensive research on objective density, future studies should prioritize the exploration of subjective crowdedness. Furthermore, the recent study on perceived crowdedness and aggression has not yet conducted an in-depth exploration of the underlying mechanisms between the two. Therefore, this study primarily focuses on perceived crowdedness, further examining its relationship with aggression and uncovering its underlying mechanisms. Apart from the aforementioned research findings, from a theoretical perspective, the relationship between perceived crowdedness and individual aggressive behavior can also be inferred. The stimulus overload theory emphasizes that in high-crowding environments where the perceptual information provided to individuals surpasses a certain stimulus threshold—an optimal arousal level and the limited information processing capacity of humans—attention can be in an overloaded state, leading to stress arousal (Andereck & Becker, 1993; Milgram, 1970). When individuals are under high levels of stress, their executive functions are further impaired (Dierolf et al., 2017; Girotti et al., 2018; Q. Liu et al., 2020), which, in turn, may lead to aggressive behavior (Holley et al., 2017). By combining theoretical perspectives and previous research findings, we infer that when individuals perceive crowdedness, they are more likely to exhibit increased aggressive behavior.

### 2.2. The Role of Relative Deprivation

Although perceived crowdedness may predict aggressive behavior, the potential mechanism remains unexplored. According to the general strain theory, the negative stimulus impacts an individual’s aggressive behavior by influencing negative emotions. Relative deprivation refers to a subjective experience characterized by negative emotions, which may serve as a mediating factor in the relationship between perceived crowdedness and aggressive behavior. Relative deprivation occurs when individuals compare themselves to a reference group and perceive themselves as disadvantaged, leading to feelings of dissatisfaction, anger, and other negative emotions (Xiong & Ye, 2016). Relative deprivation can lead individuals to engage in deviant behavior. The frustration-aggression hypothesis posits that individuals experiencing frustration will inevitably exhibit aggressive behavior, either directly or indirectly, to alleviate their frustration (Berkowitz, 1989). Previous studies have found that relative deprivation is not only immediately associated with aggression (Wang et al., 2023) but also acts as a longitudinal predictor of aggressive tendencies (Greitemeyer & Sagioglou, 2019). Furthermore, experimental research has demonstrated that individuals in conditions of relative deprivation exhibit higher levels of aggression compared to those in conditions of relative satisfaction (Greitemeyer & Sagioglou, 2017). Taken together, these theoretical perspectives and empirical findings consistently suggest that relative deprivation can positively predict individual aggressive behavior.

High levels of perceived crowdedness may increase relative deprivation. The behavioral interference theory emphasizes that when too little space, too many people, or too much social interaction prevents people from achieving their immediate goals, the obstruction of goal-directed activities can cause frustration (Bruins & Barber, 2000). Moreover, the emergence of relative deprivation must have conditions where the individual fails to achieve their goals (Shi et al., 2020). Therefore, we infer that perceived crowdedness may be associated with an increase in individuals’ relative deprivation. While no research directly illustrates the link between perceived crowdedness and relative deprivation, existing studies also provide some indirect evidence for our expected hypothesis. Properties with larger areas and better quality are valuable resources closely tied to an individual or group’s social status (X. Zhou & Suhomlinova, 2001). As an indispensable resource in people’s lives, differences in property ownership may lead to the emergence of a sense of relative deprivation for individuals or groups (Hu & Gong, 2021). Therefore, we infer that crowdedness, as a psychological representation of limited property resources, may increase relative deprivation. In summary, relative deprivation is hypothesized to mediate the relationship between perceived crowdedness and aggressive behavior.

### 2.3. The Role of Upward Social Comparison

According to the I^3^ model, impellance factors directly predict individual aggressive behavior and further interact with instigation factors, thereby influencing both aggressive behavior and its predictive factors. Upward social comparison may serve as an impellance factor. Individuals exhibit a strong tendency to compare themselves with others, and when individuals compare themselves to those they perceive as superior, it is referred to as upward social comparison (Festinger, 1954). Upward social comparison may predict aggressive behavior. Crawford (2007) found that upward social comparison can create a threat effect on individuals. To mitigate this sense of pressure or self-threat and maintain positive self-evaluation, individuals often adopt strategies for psychological compensation, and the emergence of aggressive behavior is a manifestation of the psychological need to compensate for perceived shortcomings in one’s abilities (Leander & Chartrand, 2017).

Upward social comparison, as an impellance factor, may serve a moderating role in the relationship between perceived crowdedness and relative deprivation. Upward social comparison is at the core of relative deprivation (Kim et al., 2018; Smith et al., 2012). Both cross-sectional and longitudinal studies have consistently shown that upward social comparison is a crucial predictor of individuals’ sense of relative deprivation (Buunk et al., 2003; Pan & Zhao, 2023). Through upward social comparison, members of disadvantaged groups often perceive their basic rights as being deprived or their situation being unfavorable, to a certain extent, which may damage their self-esteem (Crawford, 2007). Therefore, when facing the same sense of crowding, individuals with high upward social comparison may face damage to their self-esteem, which leads individuals to experience a greater sense of deprivation. Simultaneously, the feeling of crowdedness can activate an individual’s scarcity mindset (Fan et al., 2019). When individuals with a scarcity mindset compare themselves to others, they tend to focus more on what they lack compared to others, triggering a heightened sense of deprivation (Ren et al., 2023). As previously mentioned, a crowded living environment can impose stress on individuals, and stress increases cognitive load (Lattimore & Maxwell, 2004); a high cognitive load depletes individuals’ cognitive resources. Furthermore, the “Willpower” hypothesis suggests that individuals have an inherent tendency to engage in negative behaviors and must expend cognitive resources to overcome impulses and refrain from such behaviors (Metcalfe & Mischel, 1999). Upward social comparison can further induce cognitive load (Yan et al., 2024), thereby occupying the individual’s cognitive resources. Therefore, when combining these perspectives, when individuals’ cognitive resources are impaired by both perceived crowdedness and upward social comparison, they are more likely to exhibit aggressive behavior, which suggests that upward social comparison may play a moderating role in the relationship between perceived crowdedness and aggression.

### 2.4. The Role of Connectedness to Nature

According to the I^3^ model, inhibition factors directly predict individual aggressive behavior and further interact with instigation factors to influence aggression. Given the nature of perceived crowdedness and relative deprivation, the selection of inhibition factors in this study should consider those that alleviate stress and regulate emotions. Connectedness to nature may serve as an inhibition factor. Connectedness to nature describes an individual’s subjective sense of closeness to and bond with the natural environment, primarily reflected in the inclusion of nature in cognitive representation, emotional attachment to nature, and the desire to establish a connection with nature (Y. M. Li et al., 2018). A substantial body of research has demonstrated the positive impact of connectedness to nature on mental health. Furthermore, connectedness to nature can also predict individual behavior. When individuals feel a stronger connection to nature, they tend to act in a more environmentally friendly way (Davis et al., 2011; Tam, 2013). In other words, connectedness to nature enhances prosocial behavior while reducing aggression. Recent studies provide empirical support for this perspective, revealing that connectedness to nature negatively predicts cyberbullying, a typical form of aggressive behavior in online settings (Gao et al., 2024).

Connectedness to nature, as an inhibition factor, may serve a moderating role in the relationship between perceived crowdedness and aggressive behavior. As previously mentioned, perceived crowdedness induces stress in individuals, which, in turn, can lead to increased aggression. According to the stress-reduction theory, humans have adapted to flourish in safe, natural environments, where natural elements inherently help mitigate stress by reducing the nervous system’s response to stressors (Ulrich et al., 1991). While perceived crowdedness heightens individuals’ stress levels, connectedness to nature may help relieve this stress, indicating its role in offsetting the negative impact of perceived crowdedness. From the perspective of behavioral response patterns, individuals with a strong connectedness to nature are more frequently exposed to natural environments (Hinds & Sparks, 2008). These settings facilitate psychological restoration, help process lingering thoughts, and enhance positive emotions (Wells et al., 2019), ultimately leading to a reduction in aggressive behavior. Furthermore, connectedness to nature may also moderate the relationship between relative deprivation and aggressive behavior. This is because connectedness to nature enhances emotional regulation (Bakir-Demir et al., 2021). This suggests that when experiencing the negative emotions associated with relative deprivation, individuals with a high level of connectedness to nature are more likely to employ effective emotional regulation strategies, thereby preventing aggression triggered by negative emotions. In contrast, individuals with low connectedness to nature are more prone to adopting ineffective emotional regulation strategies, making them more likely to resort to aggression as a means of alleviating distress (Holley et al., 2017; Navas-Casado et al., 2023).

### 2.5. Research Hypothesis

**Figure 1:**
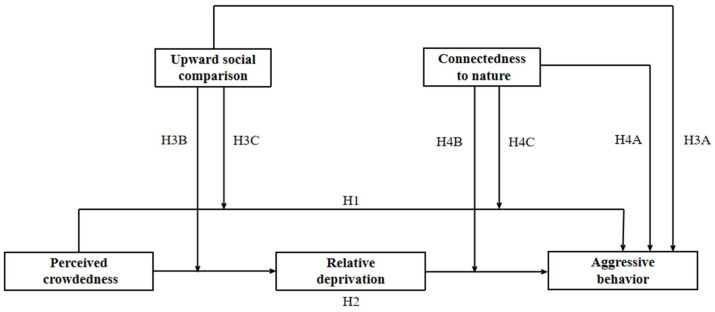
The conceptual model of this research.

Building upon the general strain theory and I^3^ model and findings from previous research, we have delineated the roles these factors (i.e., perceived crowdedness, relative deprivation, upward social comparison, and connectedness to nature) play in the impact mechanism model (Figure 1). We formulated the following four hypotheses:

**H1.**  *Perceived crowdedness may positively predict aggressive behavior.*

**H2.**  *Relative deprivation may mediate the relationship between perceived crowdedness and aggressive behavior.*

**H3A.**  *Upward social comparison may positively predict aggressive behavior.*

**H3B.**  *Upward social comparison may moderate the relationship between perceived crowdedness and relative deprivation.*

**H3C.**  *Upward social comparison may moderate the relationship between perceived crowdedness and aggressive behavior.*

**H4A.**  *Connectedness to nature may negatively predict aggressive behavior.*

**H4B.**  *Connectedness to nature may moderate the relationship between relative deprivation and aggressive behavior.*

**H4C.**  *Connectedness to nature may moderate the relationship between perceived crowdedness and aggressive behavior.*

## 3. Materials and Methods

### 3.1. Participants and Procedure

The survey was administered online, utilizing a web-based platform (Credamo, https://www.credamo.com), which is a paid research participation online platform that is widely accepted in academia. A total of 888 participants were recruited in this survey. To identify and exclude participants who may have responded inattentively, we incorporated attention check items in the survey. An example of such an item was, “For this question, please choose ‘Somewhat Agree’”. After excluding participants who failed the attention check items, the final sample consisted of 848 participants (with an effective response rate of 95.49%), including 524 (61.8%) females. The average age of the participants was 33.15 years (*SD* age = 6.83), ranging from 20 to 75 years old. A set of self-reported questions was administered to all participants. After completing the survey, participants were given 10 RMB as financial compensation.

### 3.2. Measures

#### 3.2.1. Perceived Crowdedness

The Perceived Crowdedness Scale (J. Liu et al., 2023) is a 9-item self-report scale used to measure participants’ perceived crowdedness in their living environment, and one sample item is “The neighborhood where I live and the surrounding areas always feel crowded”. Each item uses a 7-point Likert-type scale ranging from 1 (“strongly disagree”) to 7 (“strongly agree”). In our study, the internal consistency of the total score was *α* = 0.908.

#### 3.2.2. Relative Deprivation

Personal Relative Deprivation Scale (PRDS) (Callan et al., 2011) is a 5-item self-report scale. The PRDS is translated and tested on Chinese college students, but exploratory factor analyses revealed that the two reverse-worded items from the original PRDS did not load onto the primary factor for the Chinese-translated PRDS. A reduced 3-item Chinese PRDS showed good convergent validity (Peng et al., 2021). PRDS-3 was used to measure participants’ relative deprivation, and one sample item is “When I compare what I have to those who are similar to me, I feel deprived”. Each item uses a 6-point Likert-type scale ranging from 1 (“totally inconsistent”) to 6 (“totally consistent”). In our study, the internal consistency of the total score was *α* = 0.802.

#### 3.2.3. Upward Social Comparison

The upward social comparison sub-scale of the Social Comparison Scale (Gibbons & Buunk, 1999) is a 6-item self-report scale used to measure participants’ degree of upward social comparison, and one sample item is “In my daily life, I often like to compare myself with those who do better than myself”. The adaptability of the scale in the Chinese population has been verified (Bai et al., 2013). Each item uses a 5-point Likert-type scale ranging from 1 (“totally inconsistent”) to 5 (“totally consistent”). In our study, the internal consistency of the total score was *α* = 0.956.

#### 3.2.4. Connectedness to Nature

Connectedness to Nature Scale (Mayer & Frantz, 2004) is a 14-item self-report scale used to measure participants’ degree of connectedness to nature, and one sample item is “I often feel one with nature”. The adaptability of the scale in the Chinese population has been verified (N. Li & Wu, 2016). Participants were asked to indicate their level of agreement with the statements on a 5-point Likert-type scale ranging from 1 (“Strongly disagree”) to 5 (“Strongly agree”). In our study, the internal consistency of the total score was *α* = 0.619.

#### 3.2.5. Aggressive Behavior

Buss-Perry Aggression Questionnaire (Buss & Perry, 1992) is a 29-item self-report scale used to measure aggression, and one sample item is “Sometimes my friends think I’m a reckless person”. Participants were asked to indicate their level of agreement with the statements on a 7-point Likert-type scale ranging from 1 (“Strongly disagree”) to 7 (“Strongly agree”). In our study, the internal consistency of the total score was *α* = 0.926.

#### 3.2.6. Covariates

Following previous studies on prosocial behavior (Eagly & Steffen, 1986; Greitemeyer & Sagioglou, 2016; J. Liu et al., 2013), we included the following demographic variables, which may influence aggressive behavior as control variables: age, gender, and perceived socioeconomic status.

### 3.3. Data Analysis

Prior to conducting the analysis, the data were meticulously organized, coded, and entered into SPSS 24.0. Harman’s single-factor test was conducted to test for common method bias. The normality of the data was assessed by considering skewness and kurtosis scores (D’agostino et al., 1990; Kline & Santor, 1999). Following the review of the observed scale characteristics and correlations, we used Model 4 to explore the mediating effect of relative deprivation and used Model 29 to carry out a moderated mediation model to explore the effect of upward social comparison and connectedness to nature using PROCESS macro ver3.2. All variables were standardized. Furthermore, a bootstrap method with 5000 resamples was employed to estimate the 95% confidence interval (CI), allowing for the assessment of the significance of indirect effects (Hayes, 2017).

## 4. Results

### 4.1. Preliminary Analysis and Correlational Analysis

To reduce common method bias due to self-reporting, this study controlled for it procedurally and statistically. In terms of procedures, this study used anonymous surveys and reverse scoring of some items to carry out certain controls; in terms of statistics, we examined common method variance. Harman’s single-factor test found that a total of 14 factors had eigenvalues greater than 1, and the first variance explanation rate was 24.280%, which was less than the critical value of 40%, indicating no significant common method bias in this study (H. Zhou & Long, 2004).

The findings from the observed scale characteristics indicated that the kurtosis scores were between −1.484 and 2.581, and the skewness values ranged from −0.850 to 1.481, indicating that all measures had a relatively normal distribution (Table 1).

### 4.2. Correlational Analyses

The correlations between the variables are shown in Table 2. Perceived crowdedness showed positive correlations with relative deprivation (*r* = 0.460, *p* < 0.001) and aggressive behavior (*r* = 0.461, *p* < 0.001), negative correlations with connectedness to nature (*r* = −0.469, *p* < 0.001), but the correlation between perceived crowdedness and upward social comparison was not significant (*r* = 0.063, *p* = 0.066); upward social comparison showed positive correlations with relative deprivation (*r* = 0.229, *p* < 0.001) and aggressive behavior (*r* = 0.334, *p* < 0.001), but the correlation between upward social comparison and connectedness to nature was not significant (*r* = −0.034, *p* = 0.328); relative deprivation showed negative correlations with connectedness to nature (*r* = −0.296, *p* < 0.001) and a positive correlation with aggressive behavior (*r* = 0.612, *p* < 0.001); connectedness to nature showed a negative correlation with aggressive behavior (*r* = −0.341, *p* < 0.001).

### 4.3. Testing the Moderated Mediation Model

This study controlled for gender, age, and perceived socioeconomic status as variables in the data analysis. Before testing the hypothetical model, the data were tested to see if the assumption of collinearity was satisfied. Since the highest variance inflation factor value for the variables was 1.690, the multi-collinearity was not a concern.

First, Model 4 was applied to investigate the mediating role of relative deprivation in the relationship between perceived crowdedness and aggressive behavior. The findings demonstrated that perceived crowdedness had a significant positive effect on relative deprivation (*a* = 0.336, *SE* = 0.033, *p* < 0.001). In the regression equation, both perceived crowdedness and relative deprivation simultaneously predicted aggressive behavior. Specifically, perceived crowdedness showed a significant positive association with aggressive behavior (*c*’ = 0.347, *SE* = 0.032, *p* < 0.001), while relative deprivation also significantly and positively predicted aggressive behavior (*b* = 0.296, *SE* = 0.032, *p* < 0.001). These results confirmed that relative deprivation played a significant mediating role (*ab* = 0.099, *Boot SE* = 0.017, 95%*CI* = [0.068,0.134]). The mediating effect accounted for 22.31% of the total effect.

Second, Model 29 was adopted to explore the moderating effects of upward social comparison and connectedness to nature. This analysis required parameter estimation through two regression equations. As shown in Table 3, the key findings were as follows: (1) the interaction between perceived crowdedness and upward social comparison significantly and positively predicted relative deprivation; (2) the interaction between perceived crowdedness and connectedness to nature showed a marginally significant positive effect on aggressive behavior (*p* = 0.070).

**Figure 2:**
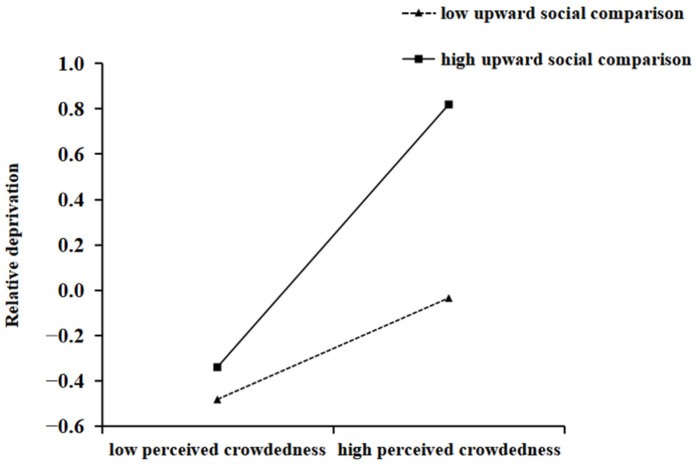
The effect of upward social comparison and perceived crowdedness on relative deprivation.

**Figure 3:**
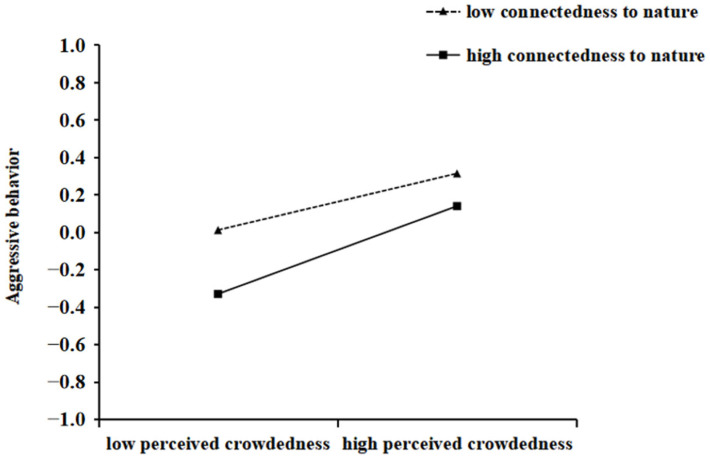
The effect of connectedness to nature and perceived crowdedness on aggressive behavior.

In order to reveal how upward social comparison moderated the impact of perceived crowdedness on relative deprivation and how connectedness to nature moderated the impact of perceived crowdedness on aggressive behavior, this study conducted a simple slope test. The total score of upward social comparison was divided into high and low groups according to the mean score plus or minus one standard deviation for difference testing. The specific results can be seen in Figure 2 and Figure 3. According to Figure 2, we concluded that in the low upward social comparison group, the predictive effect of perceived crowdedness on relative deprivation was significant (*B_simple_* = 0.224, *SE* = 0.046, *p* < 0.001); in the high upward social comparison group, the predictive effect of perceived crowdedness on relative deprivation was more significant (*B_simple_* = 0.580, *SE* = 0.043, *p* < 0.001). According to Figure 3, we concluded that in the low connectedness to nature group, the predictive effect of perceived crowdedness on aggressive behavior was significant (*B_simple_* = 0.151, *SE* = 0.046, *p* < 0.001); in the high connectedness to nature group, the predictive effect of perceived crowdedness on aggressive behavior was more significant (*B_simple_* = 0.235, *SE* = 0.043, *p* < 0.001).

## 5. Discussion

### 5.1. The Impact of Perceived Crowdedness on Aggressive Behavior and the Mediating Role of Relative Deprivation

This study investigated the association of perceived crowdedness with aggressive behavior and its internal mechanism. The results showed that perceived crowdedness was positively related to aggressive behavior, which was consistent with previous studies (Regoeczi, 2003; Weber et al., 2024); that is, perceived crowdedness may lead individuals to become more antisocial. Therefore, perceived crowdedness is an important instigation factor for aggressive behavior, which supports the general strain theory and the I^3^ model. When individuals perceive themselves to be in a crowded living environment, stressful situations persist or repeat over a long period of time, thus forming chronic stress. Chronic stress renders the long-term activation of stress response systems maladaptive (Ellis & Del Giudice, 2014). This may result in dysregulation of the hypothalamic-pituitary-adrenal (HPA) axis, which alters cortisol levels and may contribute to aggressive behavior (Mbiydzenyuy & Qulu, 2024). In addition to physiological dysregulation, stress also reduces prefrontal cortex activity, increasing impulsivity and thereby promoting aggressive behavior (Coccaro et al., 2007; Yang & Raine, 2009).

Consistent with the hypothesis, this study also showed that relative deprivation played a mediating role in the link between perceived crowdedness and aggressive behavior. This suggests that relative deprivation serves as a bridge in the process of individual social behavior. Perceived crowdedness often hinders individuals’ actions in confined spaces, not only creating pressure but also reducing their tolerance for frustration (Dai et al., 2010). Frustration is a significant psychological experience that generates more relative deprivation, and experiencing relative deprivation can trigger more negative emotional experiences (such as hostility and anger), which then lead to aggressive behavior (Greitemeyer & Sagioglou, 2019). Individuals with high levels of relative deprivation believe that they are not receiving what they deserve, leading them to be less concerned about prosocial behavior; this results in lower behavioral intensity. Our research findings also provide new insights into explaining why migrant populations tend to engage in more aggressive behavior. Due to low income and unwillingness to pay, they often cannot afford housing prices in the regular urban housing market (Zheng et al., 2009), leading to a heightened perception of crowdedness in their housing situations. This situation intensifies individuals’ perception of their low social status and the inequality in urban and rural economies, thus generating more relative deprivation (Y. Liu et al., 2019). As relative deprivation continues to increase, individuals engage in more aggressive behavior in an attempt to restore a sense of fairness.

### 5.2. The Moderating Effect of Upward Social Comparison and Connectedness to Nature

Research has found that upward social comparison plays a moderating role in the relationship between perceived crowdedness and relative deprivation. However, connectedness to nature does not moderate the relationship between relative deprivation and aggression. The risk amplification model suggests that the impact of a single risk factor is relatively limited, but when two or more risks act on an individual simultaneously, the resulting influence is much greater than the simple addition of the individual effects of these risks, potentially leading to serious consequences (Wang et al., 2023). Perceived crowdedness not only triggers a sense of deprivation in individuals, but upward social comparison also strengthens the relationship. When both factors simultaneously affect individuals, more negative effects are produced. The stress recovery and emotional regulation brought by connectedness to nature cannot offset the increased stress and negative emotions caused by crowdedness, upward social comparison, and relative deprivation. As a result, the latter half of the moderating effect of connectedness to nature in the pathway is not significant.

This study found that connectedness to nature plays a marginally significant moderating role in the relationship between perceived crowdedness and aggression. The following reasons may explain this result. First, it should be acknowledged that the reliability of the measurement tool for connectedness to nature in this study was relatively low. This may have introduced some bias in assessing individuals’ levels of connectedness to nature, making it difficult to accurately reflect their true psychological characteristics, which could further lead to the emergence of a marginal moderation effect. Second, as previously mentioned, the definition of connectedness to nature in this study encompasses both cognitive and emotional components but lacks the behavioral aspect of connection (e.g., direct contact with nature or nature exposure). Compared to cognitive and emotional connectedness to nature, behavioral connectedness to nature may be more effective in stress recovery. Additionally, some studies have confirmed the crucial role of nature exposure in emotional regulation (Ríos-Rodríguez et al., 2024). Third, we infer that the positive effects of connectedness to nature on individuals may have a “chronic” nature. In other words, although connectedness to nature can replenish the resources depleted by perceived crowdedness and, thus, reduce aggression, the restorative effects provided by connectedness to nature may take effect gradually, which may be a key explanation for the marginal moderating effect of connectedness to nature.

### 5.3. Implications

There is a lack of studies in the literature addressing the internal mechanisms between perceived crowdedness and aggressive behavior, but this study provides an empirical foundation for the field. Not only does this study (based on the general strain theory and the I^3^ model) provide a new perspective to help understand the antecedents of aggressive behavior, but it also contributes to establishing a theoretical framework for the mechanisms influencing aggressive behavior.

Second, we also provide some insights into alleviating the occurrence of aggressive behavior. From an objective environmental perspective, perceived crowdedness is a key predictor of aggressive behavior. This suggests that urban planning efforts should consider strategies to mitigate residents’ sense of crowding in densely populated living environments, which may help reduce the likelihood of aggressive behavior. Increasing green and blue spaces in crowded residential areas could be a promising approach. J. Liu et al. (2023) found that passive exposure to green and blue spaces can effectively reduce individuals’ perceived crowdedness, which, in turn, promotes prosocial behavior. Additionally, there is a need to strengthen mental health education, enhance individuals’ positive psychological qualities, and foster a more rational perspective on social comparison.

## 6. Limitations and Future Directions

Firstly, ecological systems theory posits that families and communities are the immediate contexts in which individuals directly engage with and experience their proximal environment. Following the classification of environments (Matheny et al., 1995), one category encompasses environments with physical characteristics, which is the focus of this study, specifically, perceived crowdedness. Another category involves environments with psycho-social aspects, which this study has not explored. Future research could place more emphasis on family or community environments with psycho-social characteristics to investigate how such variables influence individuals’ aggressive behavior. This approach would contribute to a more comprehensive understanding of the impact of psycho-social family or community environments on individual social behavior.

Secondly, the study results indicate that the moderating effect of connectedness to nature exhibits a marginally significant impact. In our discussion, we also highlighted that the benefits individuals derive from nature may be gradual and long-term, acting as a gradual process rather than an instantaneous remedy. The protective effect of connectedness to nature against the negative impacts of risk factors may have limitations. The following question arises: if various nature-related benefits accumulate, do they lead to cumulative effects? Future research could incorporate various psychological and objective variables related to nature benefits, such as positive nature exposure, normalized difference vegetation index, etc. This would allow for an exploration of the multiple moderating mechanisms of various nature-related benefits and further investigate whether the cumulative effects of nature benefits can effectively mitigate the negative impacts of risk factors.

Finally, this study primarily employed a questionnaire-based approach combined with a moderated mediation model to examine the mechanisms by which relative deprivation, upward social comparison, and connectedness to nature influence the relationship between perceived crowding and aggressive behavior. However, this methodology inherently limits causal inference between variables. Future research should employ behavioral experiments to validate the proposed model further. For example, researchers could manipulate individuals’ perceived crowding levels and tendencies for upward social comparison using instructions combined with pictures. Participants could then complete a relative deprivation questionnaire, followed by assessments of aggressive behavior using paradigms such as the hot sauce paradigm (Lieberman et al., 1999). Additionally, in applying the I^3^ model, this study only examined a subset of causal pathways. However, the I^3^ model encompasses 18 potential pathways linking instigation to outcome variables (Finkel & Hall, 2018), suggesting that aggression can arise through multiple interacting pathways. Future research could integrate other theoretical frameworks to further investigate the relationships among these variables, thereby advancing our understanding of the mechanisms underlying aggressive behavior.

## 7. Conclusions

This study established a moderated mediation model for a group of Chinese adults, providing a comprehensive understanding of how perceived crowdedness is related to aggressive behavior. The results revealed that perceived crowdedness and upward social comparison positively predicted aggressive behavior, whereas connectedness to nature negatively predicted it. Relative deprivation played a mediating role in the relationship between perceived crowdedness and aggressive behavior. Only upward social comparison exhibited a moderating effect in the relationship between perceived crowdedness and relative deprivation. Meanwhile, connectedness to nature showed a marginally significant moderating effect in the relationship between perceived crowdedness and aggressive behavior. This study offers empirical support for the link between perceived crowdedness and aggressive behavior based on the general strain theory and the I^3^ model; this can contribute to developing effective psychological interventions to reduce aggressive behavior.

## Figures and Tables

**Table 1:** Descriptive statistics.

Variable	*M*	*SD*	Skewness	Kurtosis
perceived crowdedness	2.249	0.841	1.481	2.581
upward social comparison	3.322	1.224	−0.470	−1.484
relative deprivation	2.158	0.878	1.436	2.364
connectedness to nature	4.379	0.273	−0.850	1.689
aggressive behavior	2.868	0.817	0.763	0.320

**Table 2:** Correlation results.

Variable	1	2	3	4	5
1 perceived crowdedness	1				
2 upward social comparison	0.063	1			
3 relative deprivation	0.460 ***	0.229 ***	1		
4 connectedness to nature	−0.469 ***	−0.034	−0.296 ***	1	
5 aggressive behavior	0.461 ***	0.334 ***	0.612 ***	−0.341 ***	1

Note: *N* = 848; *** *p* < 0.001.

**Table 3:** Moderated mediation effects of perceived crowdedness on aggressive behavior.

Variable	Model 1 (Criterion: RD)					Model 2 (Criterion: AB)				
	*β*	*SE*	*t*	95%*CI*		*β*	*SE*	*t*	95%*CI*	
				Lower	Upper				Lower	Upper
age	−0.005	0.004	−1.122	−0.014	0.005	0.001	0.004	0.271	−0.007	0.009
gender	−0.039	0.060	−0.649	−0.158	0.083	0.039	0.052	0.748	−0.065	0.145
PSS	−0.087	0.024	−3.694 ***	−0.133	−0.039	−0.023	0.021	−1.133	−0.063	0.019
PC	0.402	0.030	13.256 ***	0.334	0.475	0.198	0.033	6.072 ***	0.121	0.283
RD						0.434	0.031	14.212 ***	0.350	0.519
USC	0.249	0.030	8.233 ***	0.191	0.304	0.213	0.027	7.822 ***	0.159	0.265
PC × USC	0.178	0.033	5.388 ***	0.102	0.248	−0.025	0.029	−0.853	−0.099	0.044
CtN						−0.133	0.030	−4.465 ***	−0.191	−0.073
PC × CtN						0.042	0.023	1.811 ^+^	−0.007	0.097
RD × CtN						0.028	0.028	1.003	−0.047	0.101
*R* ^2^	0.291					0.476				
*F*	57.826 ***					76.037 ***				

Note: PSS = perceived socioeconomic status; PC = perceived crowdedness; RD = relative deprivation; USC = upward social comparison; CtN = connectedness to nature; AB = aggressive behavior. ^+^ *p* < 0.1; *** *p* < 0.001.

## Data Availability

The data are not publicly available due to privacy or ethical restrictions, but they will be available from the corresponding author upon reasonable request.
